# Stroke Investigative Research and Education Network: Public Outreach and Engagement

**DOI:** 10.4172/2161-0711.1000518

**Published:** 2017-04-24

**Authors:** A Singh, C Jenkins, B Calys-Tagoe, OS Arulogun, S Sarfo, B Ovbiagele, A Akpalu, S Melikam, E Uvere, MO Owolabi

**Affiliations:** Department of Public Health, KNUST Hospital, Kumasi, Ghana

**Keywords:** Stroke, Community, Engagement, Focus group discussions, Outreach, Community advisory boards, Sub-Saharan Africa

## Abstract

Stroke is becoming a leading cause of disability and death, and a major public health concern in Sub-Saharan Africa (SSA). The Stroke Investigative Research and Education Network (SIREN) seeks to comprehensively characterize the genomic, sociocultural, economic, and behavioral risk factors for stroke and to build effective teams for research to address and decrease the burden of stroke and other non-communicable diseases in SSA. One of the first steps to address this goal was to effectively engage the communities that suffer high burdens of disease in SSA. This paper describes the process of SIREN project's community engagement activities in Ghana and Nigeria. The aims of community engagement (CE) within SIREN are to: i) elucidate information about knowledge, attitudes, beliefs, and practices (KABP) about stroke and its risk factors from individuals of African ancestry in SSA; ii) educate the community about stroke and ways to decrease disabilities and deaths from stroke; and iii) recruit 3000 control research subjects to participate in a case-control stroke study. CE focused on three-pronged activities-constitution and interaction with Community Advisory Board (CABs), Focus Group Discussions (n=27) and community education and outreach programs (n=88). FGDs and outreach programs indicate that knowledge of stroke, as well as risk factors and follow-up evidence-based care is limited and often late. Almost all indicated that genetic testing could help health provider’s better treat stroke and help scientists better understand the causes of stroke. Over 7000 individuals have received education on cardiovascular risk factors and about 5,000 have been screened for cardiovascular risk factors during the outreaches. The CE core within SIREN is a first of its kind public outreach engagement initiative to evaluate and address perceptions about stroke and genomics by patients, caregivers, and local leaders in SSA and has implications as a model for assessment in other high stroke risk populations.

## Introduction

Over 80% of stroke deaths occur in all Low and middle income countries (LMICs) including in Sub Saharan Africa [1]. The burden (death and disability) of stroke in SSA will rise substantially over the next few decades as the sub-continent transitions from the current dominance by infectious conditions to chronic non-communicable diseases [1,2]. Identifying ways to mitigate the current and future societal toll of stroke must be a major public health priority in SSA. The aim of the Stroke Investigative Research and Education Network (SIREN) project is to comprehensively evaluate key environmental and genomic risk factors for stroke (and its subtypes) in SSA as well as building capacities in genomics, bio banking, phenomics, bioinformatics and biostatistics for brain research [1]. The first steps to address this goal were to effectively engage the communities that suffer high burden of stroke. Although community engagement (CE) is becoming relevant in health research activities, there are limited models for CE in health research in developing countries particularly SSA [3,4].

Community engagement (CE) is ‘the process of working collaboratively with and through groups of people affiliated by geographic proximity, special interests or similar situations to address issues affecting the well-being of those people’ [5]. It serves as a powerful vehicle to promote environmental and behavioral changes, leading to improvements in health and wellbeing of a community [6]. CE and community-engaged research, particularly community-based participatory research (CBPR), are increasingly being viewed as key to translational research and serve as a guide to the researcher in the discovery process as well as to the community in ownership and integration of research into improving health [7].

**Figure 1:**
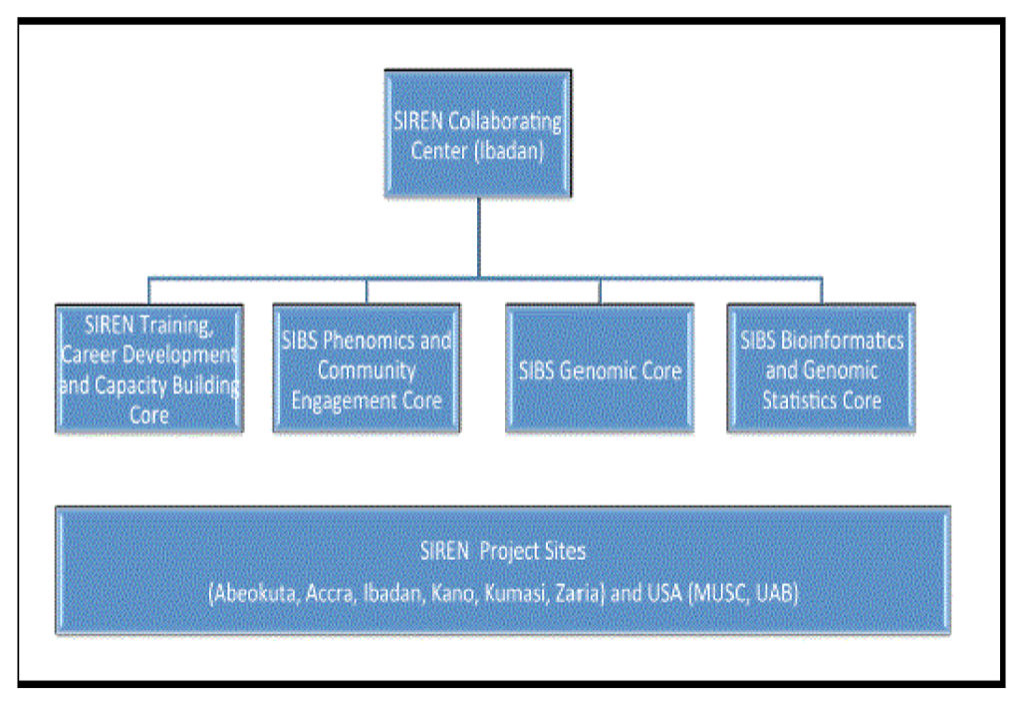
SIREN project components.

Community engagement has been integrated into SIREN (Figure 1) to facilitate community-based recruitment of almost all of the 3,000 controls (others may be recruited directly from hospitals in the community) as well as community sensitization to encourage those with stroke symptoms to seek rapid care at local hospitals to maximize stroke recovery. In an effort to reduce deaths and disabilities from stroke, there is a need to educate the community about stroke and ways to decrease disabilities and deaths from stroke using socio-culturally appropriate messaging and messengers [8]. This would allow community members to seek health care for stroke and stroke prevention in a timely manner [8]. Community engagement is also important for communities that are not familiar with phenomic and genomic studies and is also a sign of respect for values and, traditions and culture of those involved in SIREN.

Guided by two main principles: Principles of Community Engagement for the Human Heredity and Health in Africa initiative (H3Africa) [9] and Principles of Community Engagement for National Institute of Health (NIH) [10], the CE component of SIREN focused on the development and implementation of a Community Advisory Board (CAB), an exploration of the knowledge, attitudes and practices related to stroke, stroke risk factors, genetic testing, public outreach and engagement and dissemination of the results to relevant authorities and community members.

This CE core within SIREN is a novel kind of public outreach engagement initiative to evaluate and address perceptions about stroke and genomics by patients, caregivers, and local leaders (as well as health professionals) in SSA. This process along with ongoing research and translation of research findings into clinical and community behaviors and practices, will ultimately lead to improvements in stroke prevention and treatment.

There is a paucity of empirical data and published experience on community engagement related to stroke in SSA. Further research is needed for identification of authentic community representatives, methods of engagement and situations when engagement is needed [3,5,11]. This article describes how the SIREN project engaged eight sites in Ghana and Nigeria over the past three years (2014–2016), describing the community engagement activities that have arisen since inception.

### Objectives of the CE process

The aims of CE in SIREN are to illuminate information about knowledge, attitudes, beliefs, and practices about stroke and its risk factors from individuals of African ancestry in SSA, and to facilitate participation in genomic studies, as well as to help disseminate the results of SIREN. The hypothesis is that a systematic approach to community engagement using qualitative research methodologies would provide information that can be used to mobilize and educate the community regarding the nature of stroke, role of genetic testing, and benefits of involvement in research studies.

### Setting

**Figure 2:**
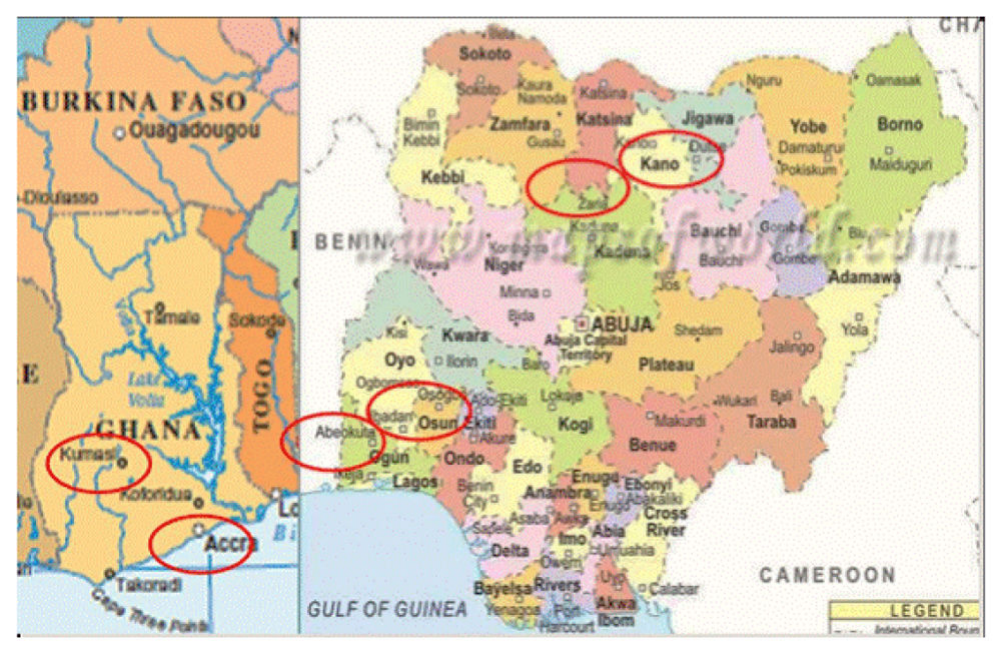
Study sites for SIREN in Ghana and Nigeria.

There are eight study sites located in six cities across Nigeria and Ghana in Western Africa. Nigeria is the most populous nation is Africa with over 177 million people in 2011 [12] (Figure 2).

As per the 2010 gross national income per capita, it was classified as a “Lower Middle Income Economy,” by the World Bank with a gross national income ranging from $1,006 to $3,975 [12]. There are over 250 ethnic groups exist with three major groups: The Hausa/Fulani (North), Yoruba (Southwest), and Igbo (Southeast); thus, providing an ideal setting for this study [1]. Six of the eight study sites are in Nigeria (two in Ibadan and two in Abeokuta, Southern Nigeria) and two in Northern Nigeria (Kano and Zaria).

There are two study sites in Ghana; a lower middle-income country in West Africa with a population of 27 million and a rapidly growing economy in Africa [1,8]. The SIREN sites are located in Accra, Southern Ghana and the other in Kumasi in middle Ghana. A brief overview of each site is presented below to provide the research and community engagement context.

## Ghana Study Sites

- Accra: The capital and largest city of Ghana with a population of 2.573 million (2011) [12]; the city was originally built as a port along the Atlantic coast and is now the economic hub of the country and recognized for the fishing, financial, and agricultural sectors [13].
- Kumasi: A city in the Ashanti Region in middle Ghana is among the largest metropolitan areas of Ghana with a population of 2.019 million (2011) [14]; it is located near Lake Bosomtwi, in a rainforest region, and is the commercial, industrial and cultural capital of Asanteman, and is called the “Garden City” because of the many beautiful plants and flowers [13].

## Nigeria Study Sites

- Ibadan: This is the capital of Oyo State and the third largest city in Nigeria. It has a population of 2.949 million and is home to the University College Hospital Ibadan [15], the first teaching hospital in Nigeria with 850 beds tertiary center. It has several community care centers including the Blossom Center for Neuro Rehabilitation which is the first neuro rehabilitation hospital in East, West and Central Africa [15].
- Abeokuta: A total population of 593,100 (16) and the Capital of Ogun State is home to the 250-bed tertiary center, Federal Medical Centre, with strong relationships to community care centers within and outside Abeokuta.
- Kano: It is the largest commercial center in northern Nigeria with fertile plains for agriculture [16] and has a population of 3.375 million. The Aminu Kano Teaching Hospital, which is a tertiary referral center with 78 beds, is in Kano.
- Zaria: The city of Zaria hosts many unique institutions of learning and is referred to as the Center of Learning in Nigeria. It has the Ahmadu Bello University Teaching Hospital with 768 beds.

### Community engagement process and structure

**Figure 3:**
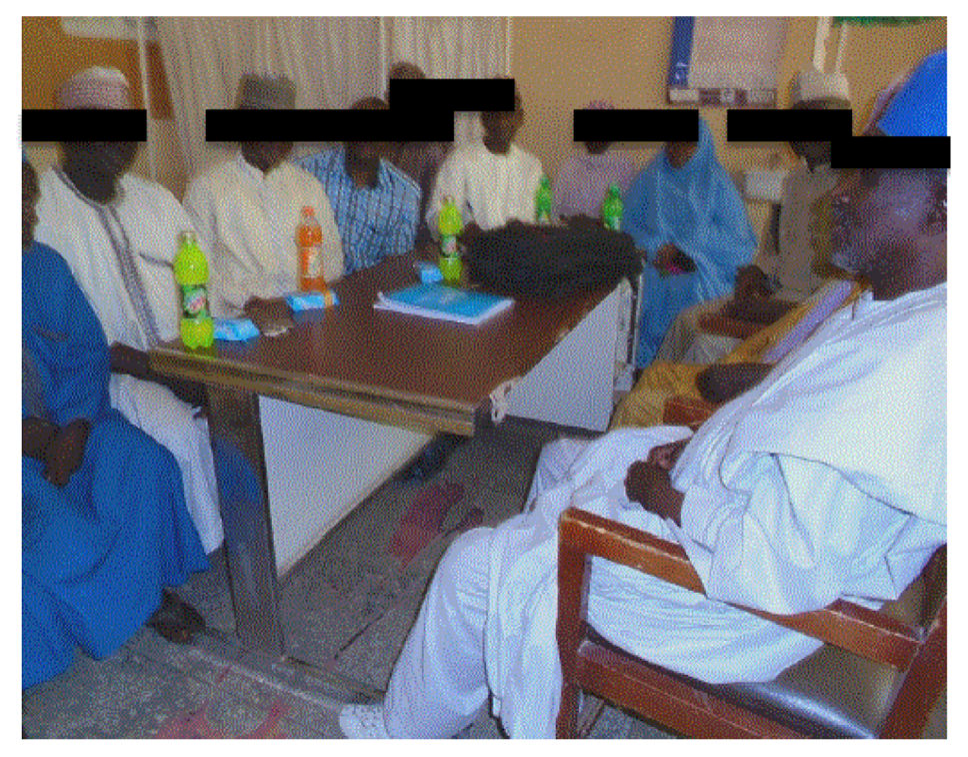
CAB meeting with Kano community engagement team in Nigeria.

Community Advisory Board (CAB): The initial engagement process in 2014 involved the formation of a community advisory board (CAB) of 6 to 9 members at each SIREN site. The membership is comprised of stroke survivor(s), faith-based leader(s), community leader(s), public health leader(s), and profession-based organizational leader(s) (Figure 3). Potential CAB members were approached by SIREN CE coordinators at each site through personal contact from an existing pool of key community members residing in SIREN study areas. A community liaison worked collaboratively with the CE coordinator at each site to identify potential CAB members. This was made possible using the Community Systems Wheel framework, which portrays the many community systems that could contribute to the success of SIREN [7].

The CABs played a key role as community liaison at each SIREN site and guides the SIREN investigators in explaining research to the local residents and facilitates the recruitment of controls as well as stroke survivors who are enrolled in the research. Initial discussions with the CAB members were to ensure that they understood the goals of SIREN so that they can communicate the goals to community members. Representatives’ were required to participate in regular quarterly community-based meetings, and ad hoc communication when needed with SIREN CE coordinator and site PI. A total of 6–10 CAB meetings at each of the various SIREN sites have been conducted during the past 3 years of SIREN (Table 1). Travel expenses are reimbursed for the quarterly meetings.

Our site specific SIREN CABs strengthened community-academic partnerships and ensured stakeholder input into operations and programs related both to community engagement and to the ongoing research processes. In addition to technical support, the CABs also provided objectivity and perspectives on generalizability in developing the overall community engagement strategies at the respective SIREN sites. The technical expertise of the CAB group at all SIREN sites brought an increased depth of understanding of the issues behind SIREN-community relations and greater coherence to the strategies developed to address them.

Standardized record keeping across SIREN sites was made possible with the following; (a) A budget worksheet for staff time allocation and expenditures for community engagement activities (b) A membership list of SIREN CABs (with name, phone numbers, address, and notes including availability, interests etc.), (c) A Calendar of Meeting for the CAB with specified date, time, location and goals and (d) A minutes record and a combined agenda.

**Figure 4:**
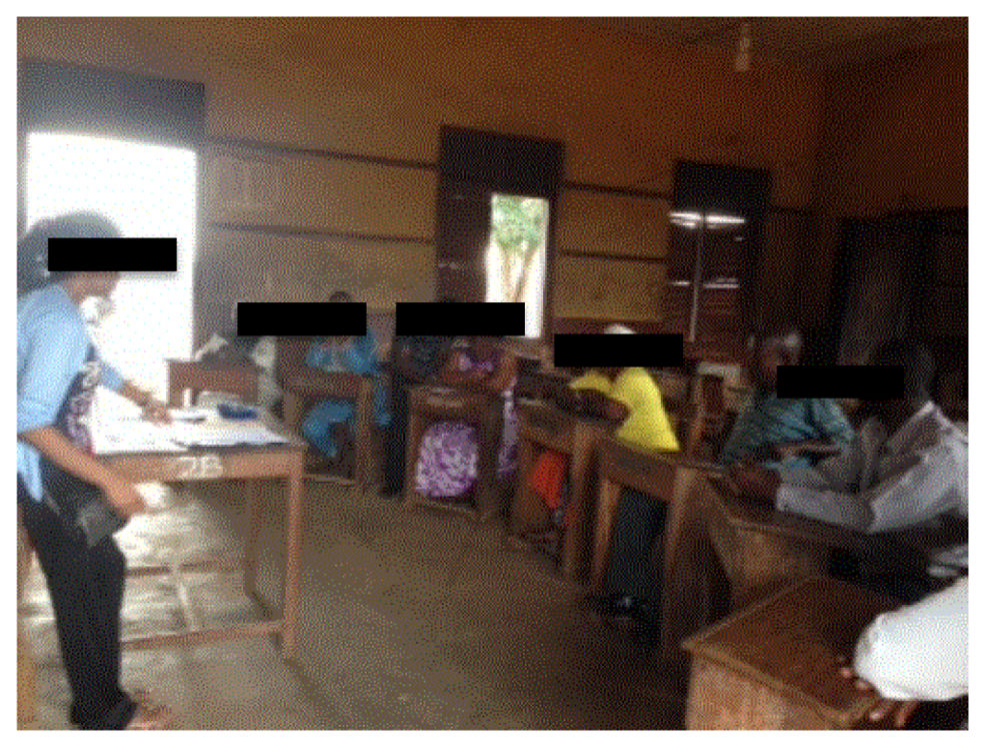
Focus group discussion among community members in Kumasi, Ghana.

Focus Group Discussions (FGDs): FGD questions were based on the theoretical framework of Arthur Kleinman’s explanatory model of illness [17], and were aimed at documenting knowledge, attitudes, and perceptions of risk factors for developing stroke, health-seeking behaviors, community attitudes, and willingness to be part of the genomic study (Figure 4).

A total of 27 focus group discussions among 168 participants including 56 health Care Providers, Community and Faith Based Leaders (n=52) and Stroke Survivors (n=60) were conducted in the 1^st^ year of the CE activities (Table 1). In contrast to community meetings, FGDs involve fewer people and target specific groups and fewer people within the larger community [18]. The findings of the FGDs have been documented in a separate paper (under review).

**Figure 5:**
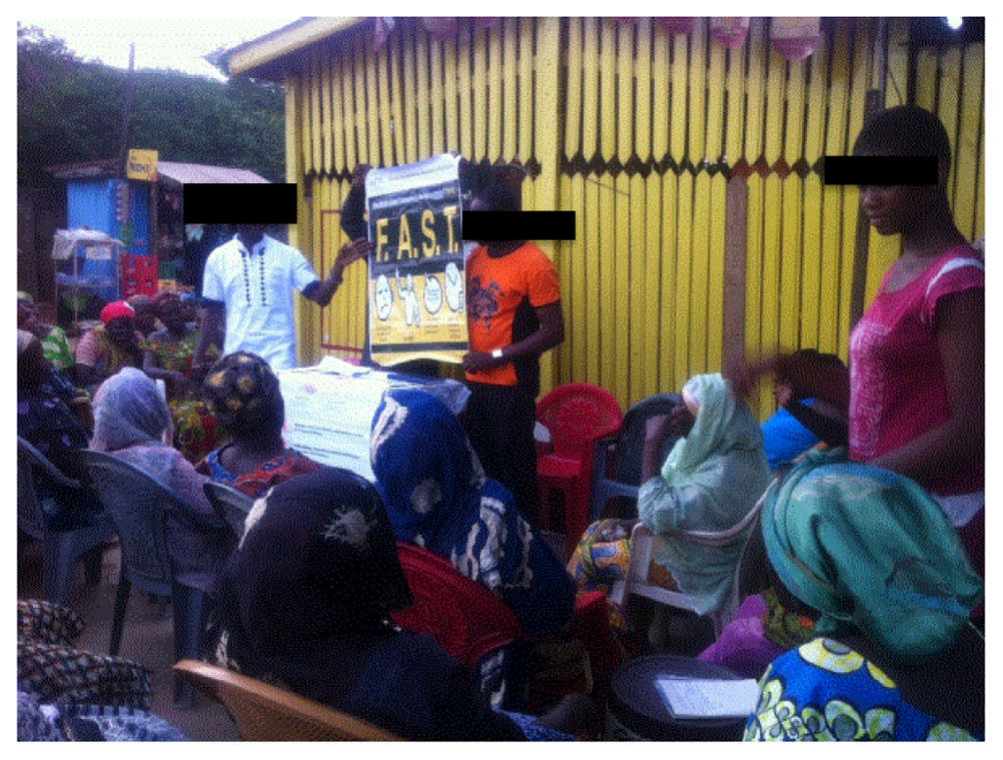
Stroke education among a Moslem women’s group in Kumasi, Ghana.

Community outreach programs and stroke education: The community engagement coordinator worked collaboratively with community groups and CAB members to provide outreach to communities and to lead community-engaged research. The CAB members activated their networks to increase recruitment when there were challenges in recruiting control subjects and assisted SIREN site CE coordinators with organizing educational events to address and share evidence-based approaches, build action-oriented community-academic research and practice partnerships, and create an impetus for community action to address stroke. The focus of the public outreach was on community-based recruitment of control subjects and the sensitization of the community to facilitate primary prevention of stroke and addressing the condition in a timely manner. The outreach was to relevant authorities, professional organization (PO), opinion leaders, faith-based organizations (such as Churches and Mosques), community organizations in an effort to introduce SIREN, its objectives, and expectations and to invite active participation and input into SIREN (Figure 5).

During the CAB meetings with the CE coordinator at each SIREN site, a number of community events and social gatherings would be identified, and with the help of the CAB, the SIREN team would incorporate a community outreach in line with on-going community activities. An example of this can be emphasized from the Kumasi site in Ghana. The Muslim community in the study site held its annual celebration of Eid-ul-fitr with an audience of close to 2000 community members mainly Moslems. The CE team in Kumasi organized an educational session on stroke during the function with the aid of visuals to the attending community members about stroke. This was a successful event and there were suggestions by community members during the feedback session to organize such programs during community events.

**Figure 6:**
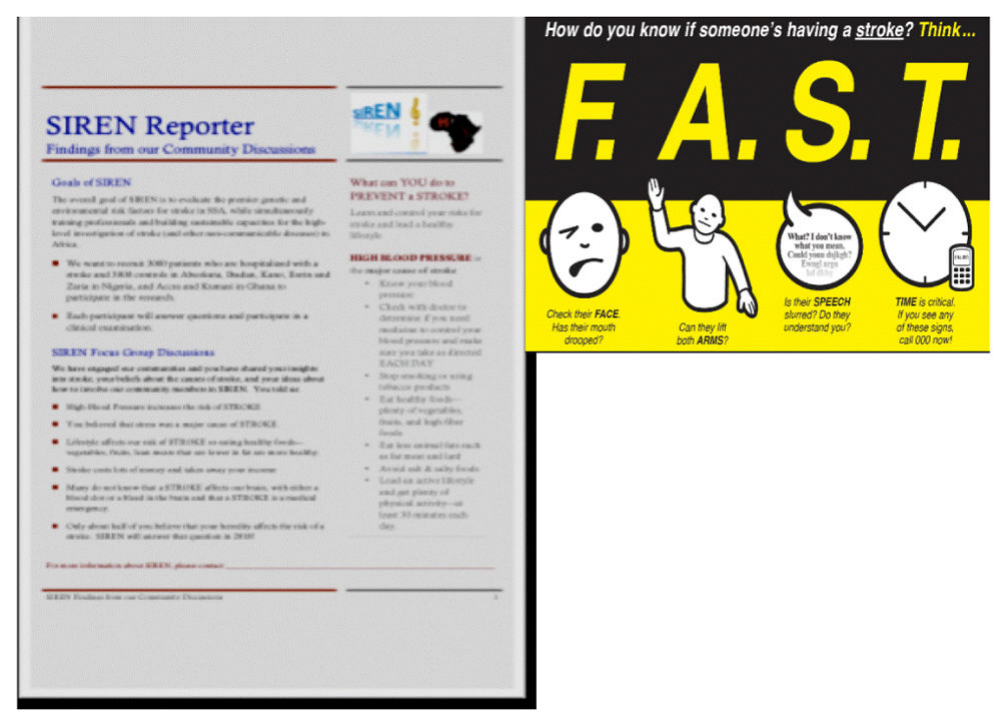
Examples of educational materials used during outreach activities.

Additionally in an effort to expand our reach into communities, the CE team developed a short video on stroke, newsletters (SIREN Reporter) and posters to educate community members about stroke and the results of their participation in the research (Figure 6).

The SIREN newsletter shares the overall SIREN Community activities, findings and culturally relevant messages for healthy lifestyles, controlling blood pressure and reducing stroke risks. The participants were also educated about stroke signs and symptoms, hypertension and diabetes and preventive actions to take at the community level. The CE team and CAB members clarified myths on stroke through the question and answer sessions at outreach programs.

A total of over 7000 individuals received education on cardiovascular risk factors and about 5000 have been screened for cardiovascular risk factors during these outreaches across all SIREN sites.

Recruitment of controls for SIREN genomics study: Tackling the burden of stroke and unraveling the reasons for its escalating epidemic requires an urgent need to determine the role of environmental and genetic factors to the risk and outcomes of stroke and its subtypes in SSA [1,19]. The aim of H3Africa, which is an international collaborative project, is to promote research in to the study of genomics and environmental determinants of common diseases with the goal of improving the health of African populations [9,20]. SIREN is similar to many of the H3Africa projects involved in the collection of human biological samples from research participants in African populations and community engagement has been identified as one of the key ethical issues by the H3Africa Working Group [9,20–22].

The genomic core of SIREN aims to explore the genetic risk factors for stroke (and its subtypes), and compare the genetic markers in the genomes of patients with Stroke which are the cases and controls which are healthy individuals from the same populations in order to look for differences between these groups that correlate with the development of stroke [1]. To be able to obtain a meaningful informed consent from study participants, information must be provided in a manner that enhances the understanding and appreciation of participants of the relevant aspects of the proposed research [23]. In studies involving participants that are less educated (such as in SIREN), having a well-written consent form does not guarantee the participants’ understanding and although the SIREN study consent forms outlined all the important aspects of the research, verbal explanations about research was required by the participants. The outreach program and the enrolment of controls at the community level provided more space for discussions about genomic aspects of the research than during recruitment of cases and controls in the hospital.

**Figure 7:**
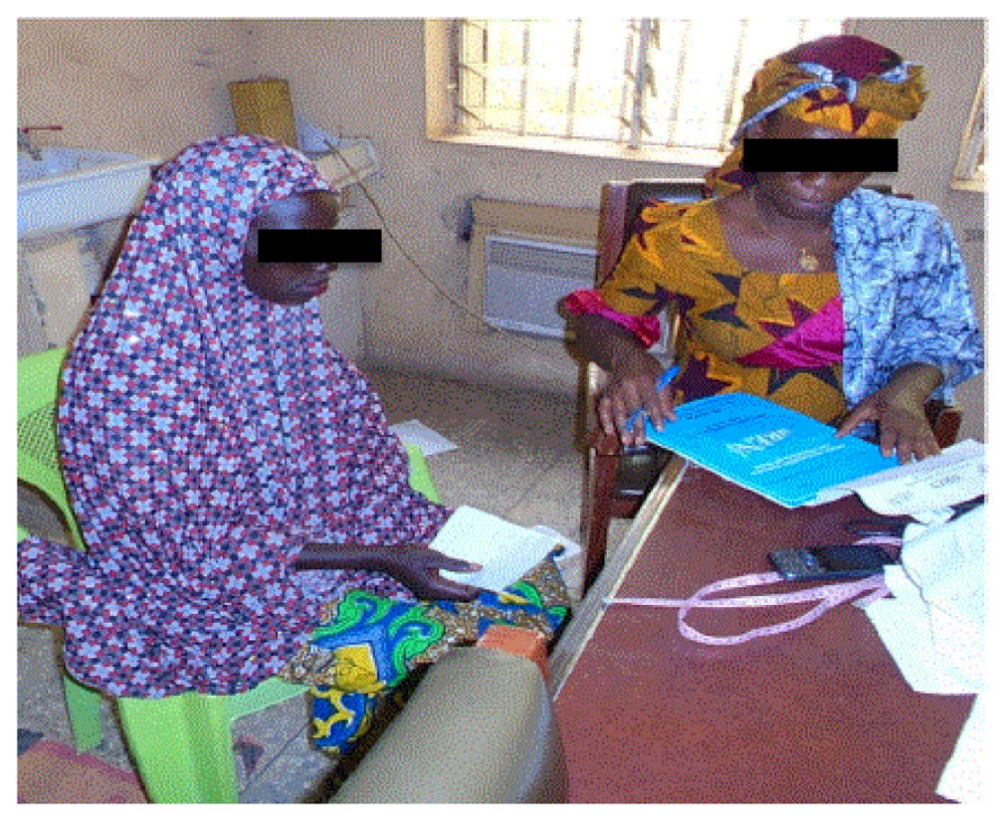
Registration of a female control during outreach activities in Nigeria.

During the outreach programs, physical examination, blood pressure, blood glucose measurements, collection of blood sample from eligible controls for storage, matching and analysis was done (Figure 7). Venous samples (35 milliliters) from community controls were obtained for sera and plasma separation for novel and emerging biomarkers of stroke and also for buffy coat preparation for DNA extraction to be used in SIBS genomics. The community controls identified were adults with no clinical evidence of stroke or myocardial infraction (MI), with or without cardiovascular risk factors matched for sex, age (± 5 years) and ethnicity. Having a known previous history of stroke, or MI, unable to provide consent and no surrogate available was an exclusion criterion for the control selection. A total of 3203 matched controls as of October 2016 have been recruited from all the eight SIREN sites so far.

## Key Opportunities and Challenges

### Opportunities

Community engagement within SIREN has provided opportunities for researchers to better understand the culture related to stroke and other diseases capture community opinions and issues related to the study, and to build community understanding of research and greater levels of trust between researchers and community members. The role of the CAB members was critical and their involvement in the development of materials fundamental in supporting informed consent processes. Community concerns and misconceptions about genomic research were addressed during the CE process and it also provided a platform for building trust between the research teams and community. A positive opinion from community members regarding research and genomic studies was received.

The CE approach in the context of genomic research provided opportunities for educating and informing about genomics and genomic research, and exchanging information between the research team and potential research participants about the research process over a period of time. The established commitment between the SIREN team and the communities at all the eight study sites has the potential to accommodate a widened scope of research beyond stroke.

### Challenges

A variety of challenges buttress the development and implementation of the community engagement strategy. One of them being the challenge of working with communities that is diverse with multiple languages and dialects spoken across and within the SIREN communities. This we tried to overcome by translating all SIREN materials from English into the appropriate languages or dialects spoken in the communities. However, translating all medical terminologies into local languages by the study team during the CE process was challenging. The average member of the broader population has only basic education and does not speak English. It was therefore crucial that the CAB members were individuals who can read and write in their own language, and who can work across diverse populations, including those with both high and low literacy, and those who truly “know their community.” Including representatives from faith-based groups in CABs was specifically beneficial to both the research team and the communities in addressing the research needs and agenda. We considered the CAB linkages to the community including their representation, technical expertise, leadership and cultural insight that each member brings to the CAB and to assure that most segments of the population are represented.

In the qualitative survey, important challenges in using qualitative methods to evaluate understanding were identified. This was mainly due to the ambiguities of the local languages around research and literacy level of the population. Developing structured questionnaires to measure understanding of research is difficult in the local context and some level of open discussion was needed to ascertain meaning.

Resources such as time, personnel, skills (facilitation, communication, negotiation, participatory training,) and funding were significant and often unpredictable. Adequate human resources such as time and skills, and flexibility were sometimes challenging for CE activities. This is due to the highly competitive nature of genetic and genomic research funding which tends to limit amounts allocated to community engagement and dissemination of research findings as well as linking activities to an agreed time line. A collaborative framework between CE coordinators, CABs and community members helped in contributing resources for many activities, such as timely planning and advertising public meetings and provision of venues and equipment. To build trust between the CAB and the SIREN research team, transparency about each individual’s motivations behind participation in the CAB during the quarterly meetings was done in addition to sharing study proposals and job descriptions with CAB members.

Other challenges including technical issues such as power cuts and access to the communities were also observed at some SIREN sites. The audiovisuals such as projectors used for the outreach programs at social gatherings required electricity sources and some of the communities did not have electricity and in places where electricity was available, there were power cuts during the shows. Access to the communities was also a challenge with some communities having poor road network, which got worse during the rainy season. There was also the challenge by the community groups not being able to distinguish research activities that included health care from routine health service delivery. The community members also suffered from participation fatigue, which increased with time and to maintain interest the research team identified counter-measures such as introducing new incentives (such as refreshments and transport costs), improving the quality of services provided community and developing new research ideas.

## Conclusion

SIREN, a multidisciplinary collaborative research, aims to enhance our understanding of factors that could be addressed to improve stroke outcomes, as well as other vascular disease entities such as coronary artery disease and chronic kidney disease in SSA; and better understand the drivers of epidemiological transition in people of African ancestry [1]. The community engagement core within SIREN is a public outreach engagement initiative to evaluate and address perceptions about stroke and genomics by patients, caregivers, and local leaders [8].

Although communicating genomics to participants and community remains a major challenge in SSA, the CE core has successfully developed a culturally appropriate CE model based on mutual understanding with community members across all study sites in Ghana and Nigeria. SIREN’s CE component provides potential contribution of community engagement to addressing ethical challenges, especially for research focused on ethnically or culturally distinct population in SSA.

Given the complexity of the goals and mechanisms, evaluation of CE initiatives is difficult. However, the SIREN CE team is in the process of evaluating the ongoing CE strategies to determine their effectiveness by various methods including estimating the coverage (number of members reached), willingness to be a part of genomic studies, ability to support the stroke study and encourage uptake of preventive measures and willingness and ability of community based organizations and faith based organizations to disseminate information among community members among others. These evaluations will assess the effectiveness and sustainability of the CE strategies described above and provides generalizable information for similar research settings over time.

SIREN CE process provides lessons for the development of CE strategies for genomic studies in Africa and provides strategies to support the early stages of a research project including recruitment of research participants. Other health research institutions in developing countries could consider these experiences. However, beyond the CE strategies mentioned in this paper, more research is required to identify effective strategies that can be used to engage research participants and their communities and find effective ways to interpret and disseminate genomic and phenomic research findings across diverse communities.

## Figures and Tables

**Table 1:** Number of FGDs, CAB members and community outreaches at SIREN sites.

Sites	Focus Group Discussions (Number of participants)	Community Advisory Members	Community Outreach Programs (n)
Accra	4 (n=30)	6	29
Kumasi	4 (n=25)	5	8
UCH	3 (n=16)	7	6
Blossom	3 (n=20)	6	6
FMC	3 (n=20)	6	16
SHH	3 (n=17)	6	2
Abuth	3 (n=19)	9	9
AKTH	4 (n=24)	7	12
Total	27 (n=168)	52	88

## References

[1] Akpalu A, Sarfo FS, Ovbiagele B, Akinyemi R, Gebregziabher M (2015). Phenotyping stroke in Sub-Saharan Africa: Stroke investigative research and education network (SIREN) phenomics protocol. Neuroepidemiology.

[2] Sarfo FS, Kyem G, Ovbiagele B, Akassi J, Sarfo-Kantanka O (2016). One-year rates and determinants of post-stroke systolic blood pressure control among Ghanaians. J Stroke Cerebrovasc Dis.

[3] Tindana PO, Singh JA, Tracy CS, Upshur RE, Daar AS (2007). Grand challenges in global health: Community engagement in research in developing countries. PLoS Med.

[4] Molyneux S, Sariola S, Allman D, Dijkstra M, Gichuru E (2016). Public/community engagement in health research with men who have sex with men in Sub-Saharan Africa: Challenges and opportunities. Health Res Policy Syst.

[5] Holzer JK, Ellis L, Merritt MW (2014). Why we need community engagement in medical research. J Investig Med.

[6] Kerasidou A (2016). Trust me, I’ma researcher!: The role of trust in biomedical research. Med Health Care Philos.

[7] Newman SD, Andrews JO, Magwood GS, Jenkins C, Cox MJ (2011). Community advisory boards in community-based participatory research: A synthesis of best processes. Prev Chronic Dis.

[8] Jenkins C, Arulogun OS, Singh A, Mande AT, Ajayi E (2016). Stroke investigative research and education network community engagement and outreach within phenomics core. Health Educ Behav.

[9] The H3Africa Consortium (2014). Enabling the genomic revolution in Africa. Science.

[10] ATSDR (2011). Principles of community engagement.

[11] McArthur-Lloyd A, McKenzie A, Findley SE, Green C, Adamu F (2016). Community engagement, routine immunization, and the polio legacy in Northern Nigeria. Global Health Communication.

[12] Central Intelligence Agency (2015). The world fact book 2014–15.

[13] Indexmundi (2013). Ghana demographics profile 2012.

[14] Nugent P (2015). Ghana in 2008.

[15] Indexmundi (2012). Nigeria demographics profile 2012.

[16] National Population Commission, ICF International (2014). Nigeria Demographic and Health Survey 2013.

[17] Kleinman A, Eisenberg L, Good B (1978). Culture, illness, and care: Clinical lessons from anthropologic and cross-cultural research. Ann Int Med.

[18] Kitzinger J (1995). Qualitative research. Introducing focus groups. BMJ.

[19] Owolabi MO, Akarolo-Anthony S, Akinyemi R, Arnett D, Gebregziabher M (2015). The burden of stroke in Africa: A glance at the present and a glimpse into the future. Cardiovasc J Afr.

[20] de Vries J, Abayomi A, Littler K, Madden E, McCurdy S (2015). Addressing ethical issues in H3Africa research–the views of research ethics committee members. Hugo J.

[21] Adoga MP, Fatumo SA, Agwale SM (2014). H3Africa: A tipping point for a revolution in bioinformatics, genomics and health research in Africa. Source Code Biol Med.

[22] Owolabi MO, Mensah GA, Kimmel PL, Adu D, Ramsay M (2014). Understanding the rise in cardiovascular diseases in Africa: Harmonising H3Africa genomic epidemiological teams and tools. Cardiovasc J Afr.

[23] Tindana P, de Vries J (2016). Broad consent for genomic research and bio banking: Perspectives from Low and Middle-Income Countries. Annu Rev Genomics Hum Genet.

